# Description of pathogenic bacteria in patients with respiratory symptoms associated with severe acute respiratory syndrome coronavirus 2 (SARS-CoV-2) in Colombia

**DOI:** 10.1186/s12941-023-00595-x

**Published:** 2023-07-07

**Authors:** Nathalia Zuluaga, David Martínez, Carolina Hernández, Nathalia Ballesteros, Sergio Castañeda, Juan David Ramírez, Marina Muñoz

**Affiliations:** 1 Centro de Investigaciones en Microbiología and Biotecnología-UR (CIMBIUR), Facultad de Ciencias Naturales, Universidad del Rosario, Bogotá, Colombia; 2 Centro de Tecnología en Salud (CETESA), Innovaseq SAS, Bogotá, Colombia; 3 Molecular Microbiology Laboratory, Department of Pathology, Molecular and Cell-Based Medicine, Icahn School of Medicine at Mount Sinai, New York, NY 10029 USA

**Keywords:** SARS-CoV-2, Co-infection, Bacterial respiratory pathogens, Respiratory symptoms

## Abstract

Viral respiratory infections may predispose to co-infections with other pathogenic microorganisms. In this study, pathogenic respiratory bacteria were detected using commercial kit Allplex^™^ Respiratory Panel 4 from nasopharyngeal samples from individuals suffering respiratory symptoms with and without severe acute respiratory syndrome coronavirus 2 (SARS-CoV-2). Patients without respiratory symptoms were included as controls. *Haemophilus influenzae* and *Streptococcus pneumoniae* were detected from 12 patients (6%) in both, patients with respiratory symptoms (including hospitalized) (n = 6) and individual without symptoms (n = 6). Pathogenic bacteria possibly proliferate due to the limited immune response of patients with SARS-CoV-2, perhaps due to dysbiosis generated by the viral infection.

**Supplementary Information:**

The online version contains supplementary material available at 10.1186/s12941-023-00595-x.

## Introduction

Since its first report in Wuhan, China, in 2019, SARS-CoV-2 has infected more than 318 million people worldwide and has approximately killed 5.5 million [1]. The symptoms described on COVID-19 disease include fever, cough, fatigue, and dyspnea, which makes it like other viral upper respiratory illnesses. The differential diagnosis for this disease can be adjusted to the patient and their comorbidities like VIH, diabetes, EPOC and others. Aside from respiratory symptoms, other symptoms identified on patients are headache, confusion, vomiting, pleurisy, sore throat, sneezing, rhinorrhea, and nasal congestion [2].

Co-infection with other pathogens is relevant since it can hinder the diagnosis, treatment, and prognosis of COVID-19 [3, 4]. These co-infections are favored due to the dysbiosis generated by SARS-CoV-2 infection, which favors the ability to increase the risk of disease progression due to impaired lung function [3, 5]. This critical situation has been reported in symptomatic patients; in most cases, coming from oral colonization by pathogenic bacteria such as *Streptococcus pneumoniae*, *Legionella pneumophila*, *Neisseria meningitidis, Moraxella catarrhalis*, among others; the last three have also been reported in cases of co-infection with influenza virus detected by multiplex PCR and mNGS analysis [5].

*Klebsiella pneumoniae*, *Haemophilus influenzae*, *Mycoplasma pneumoniae*, and *Pseudomonas aeruginosa* have been identified in SARS-CoV-2 positive samples in China, Spain, United States, Thailand, and Singapore detected by RT-PCR method. However, it is unknown how co-infection may influence symptoms severity [3, 4, 6]. Therefore, it is essential to perform studies to identify co-infections with SARS-CoV-2 and understand relationships with different epidemiological variables such as symptoms to depict the dynamics associated with COVID-19 severity.

In Colombia, several studies have reported co-infection of SARS-CoV-2 virus and other respiratory pathogens, including different bacteria, in patients from Bogotá and Cartago, respectively [7, 8]. Sánchez-Duque et al. [9] identified the simultaneous presence of up to three pathogens in Colombian samples, based on a Spanish study by Cuadrado-Payán et al. [10], or as Cataño-Correa et al. [11] call it: superinfections; of which bacteria, viruses, and fungi have been found in samples from patients with SARS-CoV-2 infection in Medellín [9, 11]. For public health purposes, it is crucial to consider the findings of this study regarding the prevalence of patients infected with SARS-CoV-2 or clinically significant respiratory bacteria. Co-infections with other potentially pathogenic agents can greatly impact diagnosis, prognosis, and treatment outcomes. In Colombia, conducting this epidemiological analysis is of utmost importance to identify population groups at high risk of acquire infection or disease progression. This information is vital for implementing appropriate monitoring strategies to alleviate respiratory symptoms exacerbated by comorbidities present in clinical diagnoses. Additionally, it provides valuable insights into the behavior and spread of the virus in Latin American countries. Therefore, the objective of this study was to identify co-infections of SARS-CoV-2 and seven potentially pathogenic bacteria associated with respiratory pathologies and correlate these findings with respiratory symptoms to better understand the dynamics associated with COVID-19 severity.

## Materials and methods

### Samples’ collection

This research corresponds to a descriptive study in which 200 nasopharyngeal respiratory samples were retrospectively analyzed. These samples were collected from patients belonging to five Colombian departments (Cundinamarca, Huila, Caquetá, Magdalena, and Atlántico) between April 6 and September 9, 2020. The patients were suspected of having COVID-19 based on the evaluation of reported respiratory symptoms. Out of the 200 samples, 100 were collected from patients who tested positive for SARS-CoV-2, while the remaining 100 were from patients who tested negative. The inclusion of samples in this study was conducted randomly. Data pertaining to these patients were documented using a standardized individual notification form for public health surveillance of acute respiratory infection caused by a new virus. This form was provided by the Ministry of Health in Colombia (Additional file 1: Fig. S1). The following age ranges were established: from 0 to 6 years (early childhood), 7 to 14 years (school age), 15 to 26 years (youth), 27 to 60 years (adulthood), and 61 years and older (elderly).We obtained ethics approval from the Research Ethics Committee of the Universidad del Rosario in accordance with the health emergency regulations outlined in Law 9-1979, decrees 786-1990, and 2323-2006.

### DNA extraction and detection of respiratory bacteria

Nucleic acids were extracted from all samples using the Hamilton Microlab Star automated system and Quick-DNA/RNA MagBead kit (Ref. R2141, Zymo Research). SARS-CoV-2 detection was performed by amplification of the E gene using primers/probe sets described in the Berlin Charité protocol [11], and the human ribonuclease P gene (RP) was detected as an internal amplification control [12]. Bacterial detection was performed using the commercial kit Allplex™ Respiratory Panel 4 (Seegene, Ref. RP9803X), following the instructions recommended by the manufacturer. This kit detects the genetic material of 7 respiratory bacteria (*Chlamydophila pneumoniae*, *Mycoplasma pneumoniae*, *Legionella pneumophila*, *Bordetella pertussis*, *Bordetella parapertussis*, *Streptococcus pneumoniae*, and *Haemophilus influenzae*) from nasopharyngeal swabs.

### Statistical analysis

A descriptive analysis of the variables, including geographical origin, gender, age, bacterial infection, symptomatology, and hospitalization, was conducted, emphasizing the respiratory symptoms of each patient. Subsequently, the frequencies of respiratory symptoms in patients with positive and negative samples were calculated and plotted. Considering that the expected values per level are less than five, a Fisher's exact test was performed to evaluate the relationship between variables. Descriptive and statistical analyses were performed utilizing R software [13]. P < 0.05 was considered statistically significant.

## Results

**Fig. 1:**
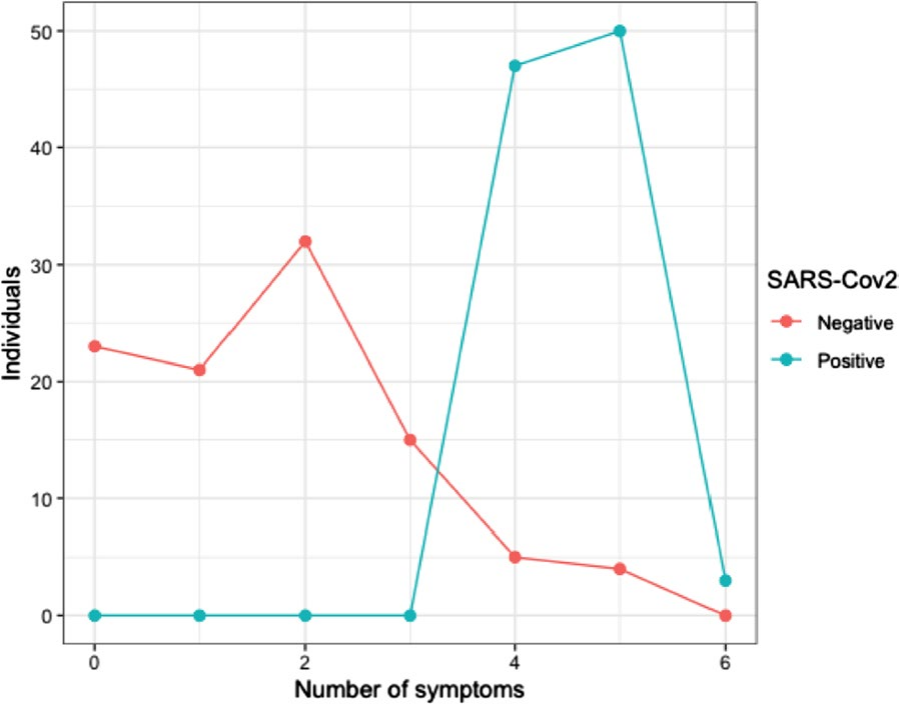
Number of individuals according to the number of symptoms they exhibit. The blue line indicates SARS-CoV-2 positive individuals, and the red line indicates SARS-CoV-2 negative individuals. Sampling was divided into two groups (i) 0 to 3 symptoms and (ii) > 4 symptoms. A Fisher’s exact test of dependence between the number of symptoms and the individuals categorized into positive and negative samples was applied, where a p < 0.05 was obtained

A total of 200 subjects were included in this study. The mean age was 44 years, with an age distribution in ranges according with the description of Table 1. A statistically significant association (p < 0.05) was evidenced between the number of symptoms and the presence of SARS-CoV-2 (Fig. 1). A positive result for SARS-CoV-2 was associated with an increase in the patients evaluated, with several symptoms greater than four. The age range with the highest frequency of respiratory symptoms was 27 years and above, of which 55.6% were positive for SARS-CoV-2. Male patients show a higher SARS-CoV-2 infection rate, several symptoms, and percentage of hospitalization compared to females.

**Table 1:** Baseline demographic characteristics and symptoms in patients included in this study

Variables	Categories	Subcategories	SARS-CoV-2		Total of individuals
			Positive (%)	Negative (%)	
Samples			100 (50)	100 (50)	200
Geographic origin					
	Cundinamarca		74 (42, 53)	100 (57, 47)	174
	Huila		15 (100)	0 (0)	15
	Caqueta		3 (100)	0 (0)	3
	Magdalene		7 (100)	0 (0)	7
	Atlantic		1 (100)	0 (0)	1
Gender					
	Male		59 (57, 84)	43 (42, 16)	102
	Female		41 (42, 84)	57 (58, 16)	98
Age ranges					
	Early childhood		1 (20)	4 (80)	5
	School age		0 (0)	3 (100)	3
	Youth		9 (30)	21 (70)	30
	Adulthood		55 (48, 25)	58 (51, 75)	114
	Elderly		34 (73, 91)	12 (26, 01)	46
	NID		1 (100)	0 (0)	1
Bacterial infection					
	*S. pneumoniae*		3 (42, 86)^*^	4 (57, 14)	7
	*H. influenzae*		2 (66,67)^*^	1 (33, 33)	3
	*S. pneumoniae*-*H. influenzae*		2 (100)^*^	0 (0)	2
Symptomatology					
	Cough		100 (67, 57)	48 (32, 43)	148
	Fever		100 (76, 34)	31 (23, 66)	131
	Odynophagia		45 (52, 33)	41 (47, 67)	86
	Rhinorrhea		11 (100)	0 (0)	11
	Respiratory distress		100 (86, 21)	16 (13, 79)	116
	Fatigue		100 (74, 63)	34 (25, 37)	134
Hospital admission					
	Hospitalized		**55 (100)**	**0 (0)**	**55**
		Male	31 (100)	0 (0)	31
		Female	24 (100)	0 (0)	24
	Non-hospitalized		**45 (31, 03)**	**100 (68, 97)**	**145**
		Male	28 (39, 44)	43 (60, 56)	71
		Female	17 (22, 97)	57 (77, 03)	74

Bold indicates presence of hospitalized patients who present one or more respiratory bacterial co-infection

**Fig. 2:**
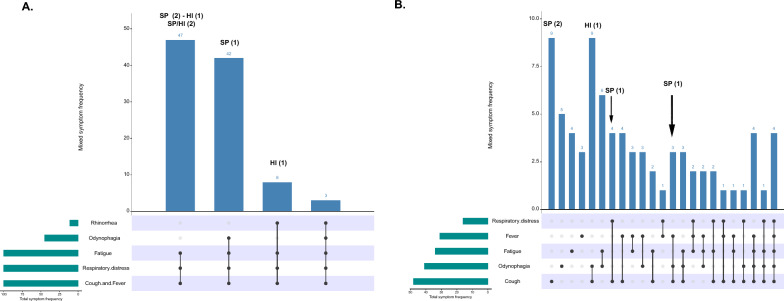
Frequency of individuals according to the combination of symptoms and their respective bacterial co-infection results. The dots indicate the combination of presenting symptoms, and the bars indicate the frequency of individuals corresponding to the combination of symptoms. **A** Positive for SARS-CoV-2 and **B** negative for SARS-CoV-2

Twelve cases of bacterial infection were identified (Table 1). Four groups of SARS-CoV-2 positive patients were identified according to the bacterial infection status and the simultaneous presence of symptoms (Fig. 2A). Patients negative for SARS-CoV-2 presented more significant variation in the simultaneous presence of symptoms (Fig. 2B). The median age of patients infected by *S. pneumoniae* and *H. influenza* bacteria was 39 years. Seven cases were reported from 27 years of age and above, including one patient with a single *S. pneumoniae* infection and six patients with the presence of SARS-CoV-2 with either *S. pneumoniae* or *H. influenza*. Patients between 0 and 26 years of age (5 individuals) presented one case of co-infection between *S. pneumoniae* and SARS-CoV-2; in the remaining four cases, the single presence of *S. pneumoniae* (three cases) and *H. influenzae* (one case) was determined.

Fifty-five hospitalized patients were reported, all with SARS-CoV-2 infection. Among these patients: two cases presented the simultaneous presence of *S. pneumoniae/H. influenzae*, while two patients had only *S. pneumoniae* and one *H. influenzae* (Table 1), there were 145 non-hospitalized patients; two patients were found with SARS-CoV-2, *S. pneumoniae*, and *H. influenzae* infection simultaneously. In addition, SARS-CoV-2 negative patients without symptoms were negative for bacterial infection.

## Discussion

Male patients show higher SARS-CoV-2 infection rate, number of symptoms and percentage of hospitalization, in relation to females. The value gap in the number of symptoms presented between men and women could also be related to the lack of resources by strict quarantine and restrictions that led mostly the male population to maintain their economic livelihood while women remained at home [14]. In addition, a typical immune response between the two sexes has been observed. In this case, the low frequency of SARS-CoV-2 infection in women may be related to a more robust and more adaptative immune response than men. Moulton [15], Channappanavar et al. [16] and Ma et al. [17] have described the significance of sex hormones in regulating molecular mechanisms within the innate and adaptive immune systems. They highlight that these hormones enhance the immune response in both male and female mice. Specifically, they emphasize the induced production of estrogen receptors as a critical factor for a competent immune response to viral infections [15–17].

This study mainly identified the simultaneous presence of *S. pneumonia* and/or *H. influenzae* with SARS-CoV-2 and infections by *S. pneumoniae* or *H. influenzae* in SARS-CoV-2 negative patients. However, no other respiratory pathogens were reported in patients exhibiting symptoms due to respiratory infection. Numerous cases of co-infection among *S. pneumoniae*, *H. influenzae*, *S. aureus*, *K. pneumoniae*, and SARS-CoV-2 have been reported in several countries [18–20]. For effective treatment of respiratory infection, it is necessary to perform the identification of the pathogen responsible for exacerbating respiratory symptoms. These findings highlight the importance of a possible well guided treatment needed to eliminate the pathogen of interest and SARS-CoV-2- avoiding the indiscriminate use of antimicrobials [21].

The main co-infection results obtained on this study were between SARS-CoV-2, *H. influenzae* and *S. pneumoniae*. The presence of *H. influenzae* can be caused by alteration of some properties of the host mucosal immunity. Consequently, this leads to failure in the control of bacterial replication due to the impact of the viral infection, generating an increase in the bacterial load in the respiratory tract [4]. Co-infection cases between SARS-CoV-2 and *S. pneumoniae* can be related to the ability of different population of this bacterium that can increase their binding rate to epithelial cells of the respiratory tract after a viral infection. Therefore, this can allow the increased production of nasopharyngeal antibodies against *S. pneumoniae* during episodes of pneumonia [4]. The simultaneous presence of *S. pneumoniae* and *H. influenzae* could be mainly due to co-colonization in cases of a weakened immune response, making them conditional pathogens [6]. Thereby, the viral infection causes alteration of host mucosal immunity properties which consequently leads to increased binding rates and bacterial load in the respiratory tract. In such case, both presence of *S. pneumoniae* and *H. influenzae* can mean a worsened host mucosal immunity not found on a single co-infection.

Subjects older than 27 years of age showed a higher rate of co-infection and symptoms related to a primary and secondary infection of respiratory pathogens (SARS-CoV-2 and one or two respiratory bacteria). This profile might be considered as an evidence that adulthood patients it is more likely to have viral infections with development of secondary oral bacterial infections that invade the respiratory microbiome, as have been previously proposed [19, 20].

Identification of SARS-CoV-2 and *S. pneumoniae, H. influenzae*, or *S. pneumoniae/H. influenzae* in hospitalized patients with a positive result for SARS-CoV-2 (Table 1) probably explains the increased number of respiratory symptoms and morbidities that compromise the prognosis of the disease [6, 22]. In France, [18] reported that these secondary pathogens are strongly associated with admission to the Intensive Care Unit (ICU) due to the need for mechanical ventilation for episodes of pneumonia in patients with co-infection. *S. aureus, S. pneumoniae, H. influenzae*, and enterobacteria were associated with secondary infection with the admission of patients to the ICU because of acute respiratory failure in patients with COVID-19 [7].

Report of SARS-CoV-2 negative patients who present a tremendous simultaneous variation of respiratory symptoms and do not show an infection by any pathogenic respiratory bacteria evaluated in this study could be associated with infection by other respiratory viruses such as Influenza virus or other members of the Coronaviridae family. A broad spectrum of symptoms typical of other types of microbial infection would be evaluated regarding the above. The National Institute of Health reports that in Colombia occur, respiratory viruses cause at least two peaks of acute respiratory infections during 2020 (the year of sample collection), which occur between March-June and September-December. This is since Colombia is a country known for its tropical climate and frequent rainy seasons typical of viral infection peaks [23].

This study has limitations regarding the limited number of samples, highlighting the need for further research that evaluates potential associations between pathogens, including at the interdomain level, in a larger and more diverse population. Furthermore, it is essential to acknowledge the limitations in the number of bacteria evaluated by the commercial kit used. Therefore, it is recommended to consider future studies that explore the composition of microbial communities from a broader perspective, such as deep sequencing of ribosomal markers or approaches using shotgun metagenomics.

In conclusion, in this study, a higher rate of SARS-CoV-2 infection was detected in men and patients older than 27 years. A co-relation was found between SARS-CoV-2 co-infection with respiratory bacteria and a higher number of respiratory symptoms. These findings characterize respiratory pathogens' behavior in Colombia patients, evidencing the need to screen different infectious agents or evaluate the entire microbial communities’ composition at a respiratory level in a complete approach to provide adequate management and treatment of symptomatic respiratory patients.

## Supplementary Information

**Additional file 1: Figure S1.** Standardized individual notification form for public health surveillance line for an acute respiratory infection due to a new virus, distributed by the Ministry of Health in Colombia.

## Data Availability

All data generated or analyzed during this study are included in this published article and its additional information files.
